# Reduction in Opioid Requirements Following Changes to Regional Anesthesia for Patients Undergoing Total Knee Arthroplasty

**DOI:** 10.31486/toj.24.0137

**Published:** 2025

**Authors:** Jeffrey Mauras, Michael McMahon, Jaudé Petrie, Ryan Roubion, Amy Bronstone, Claudia Leonardi, Vinod Dasa

**Affiliations:** ^1^ Physical Medicine and Rehabilitation Pain Medicine, Louisiana State University Health Sciences Center, New Orleans, LA; ^2^ Department of Radiology, Louisiana State University Health Sciences Center, New Orleans, LA; ^3^ Department of Psychiatry, Louisiana State University Health Sciences Center, New Orleans, LA; ^4^ Department of Orthopedic Surgery, Louisiana State University Health Sciences Center, New Orleans, LA; ^5^ Community Health Science & Policy, School of Public Health, Louisiana State University Health Sciences Center, New Orleans, LA

**Keywords:** *Analgesics*, *liposomal bupivacaine*, *multimodal pain protocol*, *opioids*, *ropivacaine*, *total knee arthroplasty*

## Abstract

**Background:**

Newer analgesic techniques to reduce opioid use and pain after total knee arthroplasty (TKA) include preoperative cryoneurolysis, adductor canal block (ACB), and local anesthetic infiltration between the popliteal artery and capsule of the knee (iPACK) block. The purpose of this study was to evaluate whether changing the regional analgesic from ropivacaine to liposomal bupivacaine would provide superior pain relief and reduce opioid requirements at 2 and 12 weeks following TKA.

**Methods:**

We conducted a retrospective medical records review of 140 consecutive patients who underwent primary TKA at a single site and received ACB with ropivacaine (multimodal-ropivacaine [MM-R] group, n=70) or ACB/iPACK with liposomal bupivacaine (multimodal-liposomal bupivacaine [MM-LB] group, n=70). The primary outcomes were the morphine milligram equivalent (MME) of filled opioid prescriptions at discharge and during the first 12 weeks after TKA, as well as the Knee injury and Osteoarthritis Outcome Score and the Patient-Reported Outcomes Measurement Information System pain intensity and pain interference scores at 2 and 12 weeks postsurgery.

**Results:**

The median MMEs for discharge opioid prescriptions and all opioid prescriptions were, respectively, 65% (*P*<0.0001) and 48% (*P*<0.0001) lower for patients in the MM-LB group vs the MM-R group. The MM-LB group had significantly better patient-reported outcomes 2 weeks after TKA compared to the MM-R group.

**Conclusion:**

Compared with ropivacaine-based regional analgesia, liposomal bupivacaine–based regional analgesia in the context of a modern multimodal pain regimen may reduce opioid requirements and improve patient-reported outcomes during acute and short-term recovery after TKA.

## INTRODUCTION

Knee osteoarthritis is one of the most common joint disorders and is a leading cause of pain and disability among adults.^1^ The definitive treatment for end-stage knee osteoarthritis is total knee arthroplasty (TKA), a procedure that has been performed with increasing frequency.^2^ An analysis of TKA procedures performed in the United States from 2009 to 2015 found an upward trend over time, with more than 600,000 procedures performed in 2015.^2^ Although the growth rate of TKA procedures has been slowing in recent years, models based on 2008 to 2014 data project that approximately 935,000 TKA procedures will be performed in the United States by 2030.^3^

Although TKA is usually successful, patients experience a considerable amount of pain during recovery that can impede early ambulation, increase hospital length of stay and health care costs, and reduce functional recovery and patient satisfaction. Oral analgesics, including opioids, have been a mainstay of pain management following TKA.^4^ However, multimodal analgesia, which uses a combination of analgesic techniques that act on pain through different mechanisms, has been shown to improve patient comfort and reduce the need for opioids that have problematic side effects and can result in physical dependence, abuse/misuse, diversion, and overdose deaths.^5^ Newer analgesic techniques that have been incorporated into multimodal analgesia in TKA include preoperative cryoneurolysis, adductor canal block (ACB), and local anesthetic infiltration between the popliteal artery and capsule of the knee (iPACK) block.

ACB involves the single or continuous injection of local anesthetic at the adductor canal. ACB is advantageous for analgesia in TKA because the block selectively disrupts sensory nerves to provide anesthesia to the anterior and medial knee,^6^ has minimal effects on motor nerves, and preserves quadriceps strength and ambulation compared with femoral nerve block.^7,8^ The 3 studies to date that have compared the efficacy of ACB with the extended-release analgesic liposomal bupivacaine vs ropivacaine or standard bupivacaine have reported mixed results during very short follow-up periods.^9-11^ A retrospective study of TKA patients who received ACB with ropivacaine or liposomal bupivacaine found significantly less opioid consumption at 8 hours postrecovery in the liposomal bupivacaine group but no statistically significant differences in opioid use or pain scores at 24 and 48 hours after surgery.^9^ In another retrospective study, TKA patients who received ACB and a periarticular injection with liposomal bupivacaine had significantly less pain and opioid consumption on postoperative days 0 and 1 and walked more than patients who received ACB and a periarticular injection with standard bupivacaine.^11^ The authors surmised that most of the benefits were attributable to the ACB with liposomal bupivacaine instead of the periarticular injection with liposomal bupivacaine because studies of periarticular injection have not shown an advantage for liposomal bupivacaine vs standard bupivacaine.^12-14^ In contrast to the findings of the retrospective studies, a randomized clinical trial that enrolled 100 TKA patients who received either ACB with ropivacaine or liposomal bupivacaine in the context of multimodal analgesia that included an iPACK blockade with ropivacaine found no statistically significant differences in opioid use or in pain and function scores during the first 72 hours after surgery.^10^

The iPACK block is an analgesic technique intended to provide effective analgesia for posterior knee pain following TKA.^15^ Recent (2021) systematic reviews of studies that have compared ACB alone vs ACB and iPACK indicate that the addition of iPACK modestly improves pain 12 hours after TKA but has no apparent subsequent benefit.^16,17^

Convincing evidence showing that regional analgesia with extended-release liposomal bupivacaine results in better pain control than regional analgesia using traditional analgesics (eg, ropivacaine) following TKA is lacking. The purpose of this retrospective cohort study was to evaluate the effects of changing the regional analgesic from ropivacaine to liposomal bupivacaine in the context of multimodal analgesia that included preoperative cryoneurolysis. Principal outcomes of interest were opioid prescribing and patient-reported outcomes during the first 2 weeks and 12 weeks following TKA.

## METHODS

### Patient Selection and Study Design

This retrospective cohort study was approved by the Louisiana State University Health Sciences Center–New Orleans Institutional Review Board and included all consecutively treated patients who underwent primary unilateral TKA performed by a single surgeon (VD) at a private academic hospital between March 1, 2017, and January 31, 2019. One study group comprised consecutively treated patients who underwent TKA between March 1, 2017, and March 18, 2018, and received a standard multimodal pain protocol consisting of preoperative cryoneurolysis and ACB with ropivacaine (the multimodal-ropivacaine [MM-R] group). The second group comprised consecutively treated patients who underwent TKA between March 19, 2018, and January 31, 2019. For this group (the multimodal-liposomal bupivacaine [MM-LB] group), the regional analgesic was changed from ropivacaine to liposomal bupivacaine for the ACB, and a liposomal bupivacaine–based iPACK was added as a combined strategy to improve the multimodal analgesia regimen; other elements of the multimodal pain regimen remained the same.

### Surgical Technique and Follow-Up

All patients underwent computer-assisted navigated TKA using an anterior midline incision and medial parapatellar arthrotomy. The surgical technique was the same for all patients. The surgeon selected either cemented or uncemented components using the NexGen Complete Knee Solution (Zimmer Biomet Holdings, Inc) or the Persona Knee (Zimmer Biomet Holdings, Inc) total knee implant systems. For cemented TKA, the tourniquet was only used at the time of cementation; no tourniquet was used in uncemented TKA cases. Patients were seen for in-office follow-up visits at 2 weeks and 12 weeks following surgery.

### Multimodal Pain Protocols

Table 1 summarizes the components of the multimodal pain protocol used for each treatment group.

**Table 1.** Multimodal Pain Protocol by Treatment Group

	Multimodal Protocol Components				
Treatment Group	Preoperative	Intraoperative Systemic	Intraoperative Regional	Postoperative Inpatient	Postoperative Outpatient
MM-R	Cryoneurolysis	Intravenous dexamethasone 10 mg and tranexamic acid 1 g	ACB with 20 mL ropivacaine 0.2%, dexamethasone 1 mg, clonidine 25 μg, and epinephrine 1:200,000	Oral acetaminophen 650 mg TID, oral celecoxib 200 mg BID, pregabalin 150 mg BID, and oxycodone 10 mg prn	Oral acetaminophen 650 mg Q 6 hrs, dexamethasone 75 mg BID, and oxycodone 5 mg prn^a^
MM-LB	Cryoneurolysis	Intravenous dexamethasone 10 mg and tranexamic acid 1 g	ACB with 5 mL injection of liposomal bupivacaine 133 mg/10 mL and iPACK with 10 mL injection of liposomal bupivacaine 133 mg/10 mL admixed with bupivacaine HCL 0.25%	Oral acetaminophen 650 mg TID, oral celecoxib 200 mg BID, pregabalin 150 mg BID, and oxycodone 10 mg prn	Oral acetaminophen 650 mg Q 6 hrs, dexamethasone 75 mg BID, and oxycodone 5 mg prn^a^

^a^From January 20, 2018, through study end on January 31, 2019, the initial prescription of oxycodone was changed 5 times. The discharge prescriptions were 84 pills of oxycodone 5 mg from March 1, 2017, to January 19, 2018; 42 pills of oxycodone 5 mg from January 20, 2018, to March 31, 2018; 30 pills of oxycodone 5 mg from April 1, 2018, to July 31, 2018; 19 pills of oxycodone 5 mg from August 1, 2018, to September 18, 2018; 28 pills of oxycodone from September 19, 2018, to December 4, 2018; and 42 pills of oxycodone 5 mg from December 5, 2018, to January 31, 2019.

ACB, adductor canal block; BID, twice daily; HCL, hydrochloride; iPACK, local anesthetic infiltration between the popliteal artery and capsule of knee block; MM-LB, multimodal-liposomal bupivacaine; MM-R, multimodal-ropivacaine; prn, as needed; Q 6 hrs, every 6 hours; TID, thrice daily.

#### Preoperative Cryoneurolysis.

Five days before surgery, all patients received preoperative cryoneurolysis targeting the infrapatellar branch of the saphenous nerve and the anterior femoral cutaneous nerve that innervate the anterior aspect of the knee.^18^ Cryoneurolysis, performed using the iovera° system (Pacira Pharmaceuticals, Inc),^19^ is a minimally invasive procedure in which a handheld device is used to apply temperatures from –100 °C to –20 °C to peripheral sensory nerves, causing Wallerian degeneration of the nerve axons and a long-acting nerve block.^20,21^ During this process, the endoneurium and myelin sheath are left intact, allowing regeneration of the nerve.^22^ Preemptive analgesia administered before cryoneurolysis included a single dose of acetaminophen 650 mg, pregabalin 300 mg, and celecoxib 200 mg.

#### Regional Analgesia.

**Figure 1.**
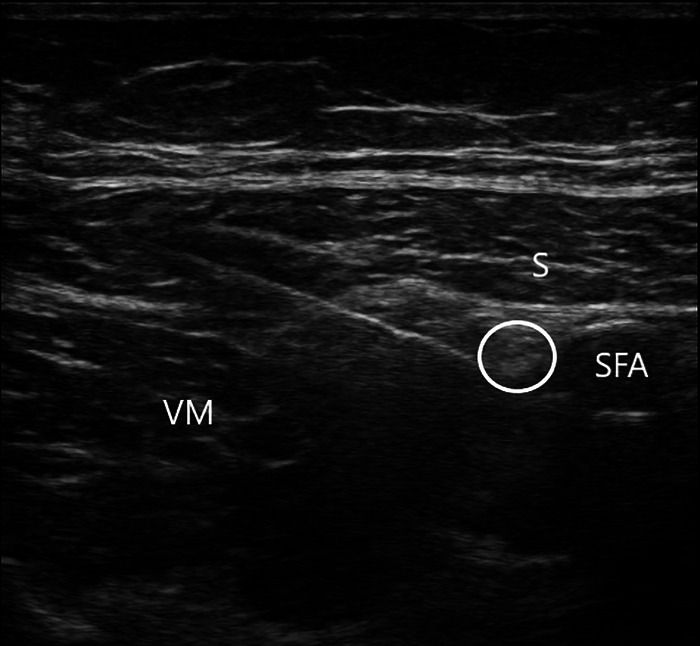
In this image of an ultrasound-guided adductor canal block, the needle can be seen entering from the lateral side between the sartorius (S) and the vastus medialis (VM) muscles. The target is the circled saphenous nerve that is just lateral to the superficial femoral artery (SFA).

Approximately 30 to 45 minutes before surgery, all patients received an ultrasound-guided ACB (Figure 1), but the MM-R and MM-LB groups received different regional analgesia.

Prior to March 19, 2018, the ACB for the MM-R group consisted of 20 mL ropivacaine 0.2%, dexamethasone 1 mg, clonidine 25 μg, and epinephrine 1:200,000. No iPACK block was performed for the MM-R group.

**Figure 2.**
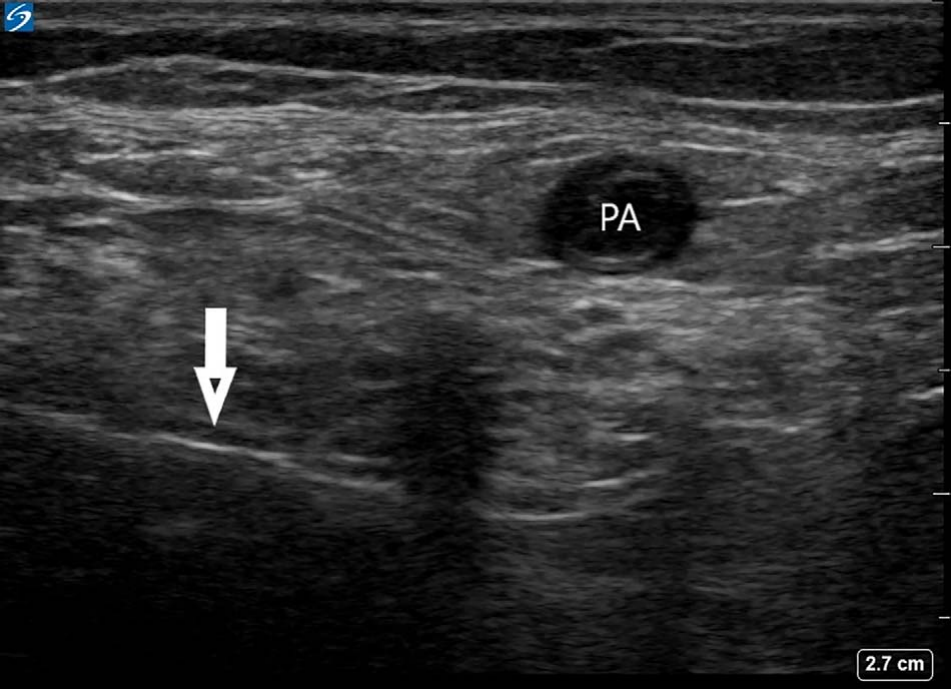
In this image of an ultrasound-guided local anesthetic infiltration between the popliteal artery (PA) and capsule of the knee (iPACK) block, the needle (arrow) can be seen entering from the lateral side into the space between the PA and the femur (out of frame to the right).

Beginning March 19, 2018, the MM-LB group received the new regional analgesia strategy that included an ultrasound-guided ACB consisting of a single 5 mL injection of liposomal bupivacaine 133 mg/10 mL admixed with bupivacaine hydrochloride (HCL) 0.25% and an iPACK block (Figure 2) consisting of a single 10 mL injection of liposomal bupivacaine 133 mg/10 mL admixed with bupivacaine HCL 0.25%.

#### Systemic Analgesia.

All patients in the MM-R and MM-LB groups received intravenous dexamethasone 10 mg and tranexamic acid 1 g intraoperatively. Postoperatively, all patients in both treatment groups received acetaminophen 650 mg every 8 hours, celecoxib 200 mg twice daily, pregabalin 150 mg twice daily, and oxycodone 10 mg every 6 hours as needed while in the hospital. Upon discharge, patients were instructed to take acetaminophen 650 mg every 6 hours and dexamethasone 75 mg every 12 hours.

#### Discharge Opioid Prescribing.

The standard discharge opioid prescription was reduced during the study period based on the observation that pain control was improved following the changes to regional anesthesia. During the study period (from January 2018 to January 2019), the standard discharge prescription was changed 5 times. The discharge prescriptions were 84 pills of oxycodone 5 mg (630 morphine milligram equivalent [MME]) from March 1, 2017, to January 19, 2018; 42 pills of oxycodone 5 mg (315 MME) from January 20, 2018, to March 31, 2018; 30 pills of oxycodone 5 mg (225 MME) from April 1, 2018, to July 31, 2018; 19 pills of oxycodone 5 mg (140 MME) from August 1, 2018, to September 18, 2018; 28 pills of oxycodone (210 MME) from September 19, 2018, to December 4, 2018; and 42 pills of oxycodone 5 mg (315 MME) from December 5, 2018, to January 31, 2019. Although the overall trend was a reduction in the amount of opioids prescribed in the standard discharge prescription, periodic increases and decreases to the discharge opioid prescription were made based on patient feedback, requests to the surgeon for more opioids, and increased requests for refills.

Patients could request an opioid prescription refill at the 2-week follow-up visit. The standard opioid refill prescription was consistent throughout the study and consisted of 90 pills of tramadol 50 mg (900 MME). At the surgeon's discretion, the standard discharge and refill opioid prescriptions could be increased (eg, for patients with preoperative opioid tolerance or low pain tolerance) or decreased (eg, for patients who were sensitive to opioid-related side effects or requested fewer opioids).

### Data Sources for Outcome Measures

Patient demographics and clinical characteristics were derived from medical records. The Knee injury and Osteoarthritis Outcome Score (KOOS) and scores for the Patient-Reported Outcomes Measurement Information System (PROMIS-29 Profile v10) were collected prior to surgery and at postoperative follow-up visits at 2 weeks and 12 weeks. Raw scores for the KOOS subscales of symptoms, pain, function in activities of daily living, and quality of life were converted to a 0 to 100 scale, with 0 indicating extreme knee problems and 100 indicating no knee problems.^23^ Although the PROMIS-29 assesses 7 health domains, only the PROMIS-29 pain intensity and pain interference scores were included in our study. The pain intensity score is derived from a single item assessing pain intensity on a 0 to 10 numeric rating scale, with 0 indicating no pain and 10 indicating the worst pain imaginable. Pain interference scores were derived from the 4-item PROMIS-29 pain interference domain and are reported using T-scores, with 50 representing the mean of the reference population and 10 as the standard deviation.^24^ A higher T-score indicates greater pain interference, meaning pain has a more substantial impact on daily activities.

Data on opioid prescriptions filled during the 12 weeks after TKA were obtained from the Louisiana Board of Pharmacy Prescription Monitoring Program.^25^ We calculated the MME for the initial prescription, the total MME for the refill prescriptions, and the total MME for the initial and refill prescriptions during the 12-week follow-up.

### Statistical Analysis

Data were analyzed using SAS/STAT software version 9.4 (SAS Institute Inc). Baseline variables by treatment group were compared using the chi-square test for categorical variables and a general linear model for continuous variables. Median MMEs were compared between groups using the nonparametric Mann-Whitney *U* test. Regression analyses were conducted to determine which patient demographics and clinical characteristics were related to preoperative patient-reported outcomes and should be covariates in subsequent multivariable analyses of patient-reported outcomes. Age was the only statistically significant predictor of preoperative patient-reported outcomes and therefore was the only covariate included in the multivariable regression analyses. The effects of group, time, and the interaction between group and time on postoperative KOOS subscale scores and PROMIS-29 pain intensity and pain interference scores were evaluated using repeated measures analysis of covariance that adjusted for age and preoperative scores. When a significant treatment effect was found, groups were compared at each time point using the *t* test. Length of hospital stay was compared using the chi-square test. A two-sided test with *P*<0.05 indicated statistical significance.

A total of 140 subjects would be needed (70 per group) to detect a clinically relevant difference of 10 points between the 2 groups on the KOOS subscales with 80% power and α=0.05, assuming a 10% loss to follow-up at 12 weeks.

## RESULTS

### Sample Characteristics

A total of 140 patients were included in the study (MM-R group, n=70; MM-LB group, n=70). No statistically significant differences were found between groups on any demographic or preoperative clinical variable (Table 2). The overall sample was predominantly female (70%; n=98) and White (60%; n=84), with a mean age of 68.8 years and mean body mass index of 32.0 kg/m^2^. Most patients had either private insurance (35.0%) or Medicare Advantage (35.0%), with 25.0% insured by Medicare and 5.0% by Medicaid.

**Table 2.** Patient Demographics and Preoperative Clinical Characteristics

Variable	MM-R Group, n=70	MM-LB Group, n=70	*P* Value
Age, years, mean ± SD	68.9 ± 9.2	68.7 ± 7.9	0.899
Sex			0.269
Male	18 (25.7)	24 (34.3)	
Female	52 (74.3)	46 (65.7)	
Body mass index, kg/m^2^, mean ± SD	31.3 ± 4.8	32.6 ± 5.1	0.133
Race			0.626
Black or African American	25 (35.7)	27 (38.6)	
White or Caucasian	44 (62.9)	40 (57.1)	
Other	1 (1.4)	3 (4.3)	
Insurance type			0.747
Private	22 (31.4)	27 (38.6)	
Medicare	20 (28.6)	15 (21.4)	
Medicaid	4 (5.7)	3 (4.3)	
Medicare Advantage	24 (34.3)	25 (35.7)	
Kellgren-Lawrence grade			1.000
3	3 (4.3)	2 (2.9)	
4	67 (95.7)	68 (97.1)	
Laterality			0.398
Right	37 (52.9)	32 (45.7)	
Left	33 (47.1)	38 (54.3)	
Charlson Comorbidity Index, mean ± SD	3.2 ± 1.8	3.3 ± 1.9	0.782
Overall deformity, degrees, mean ± SD	8.0 ± 4.3	7.7 ± 4.5	0.615
KOOS subscale, mean ± SD			
Symptoms	42.1 ± 18.7	42.6 ± 22.8	0.893
Pain	39.9 ± 18.0	40.8 ± 20.6	0.793
Function in activities of daily living	41.4 ± 19.9	41.9 ± 20.1	0.904
Quality of life	24.1 ± 18.2	23.5 ± 19.7	0.847
PROMIS-29 score, mean ± SD			
Pain intensity	7.3 ± 2.0	7.2 ± 2.2	0.843
Pain interference	64.7 ± 6.9	64.9 ± 8.0	0.899

Note: Data are reported as n (%) unless otherwise indicated.

KOOS, Knee injury and Osteoarthritis Outcome Score; MM-LB, multimodal-liposomal bupivacaine; MM-R, multimodal-ropivacaine; PROMIS-29, Patient-Reported Outcomes Measurement Information System.

### Opioid Prescribing

Table 3 compares opioid prescribing by treatment group. The median MMEs for the initial opioid prescription and for all opioid prescriptions were, respectively, 65% (*P*<0.0001) and 48% (*P*<0.0001) lower for the MM-LB group vs the MM-R group. The reduction in the initial opioid prescription MME is attributable to a change in the surgeon's opioid prescribing practice over the course of the study that affected 4 patients in the MM-R group and all patients in the MM-LB group. No statistically significant differences were found between groups for the percentage of patients with at least 1 refill, the median number of refills, or the median total MME for refill prescriptions.

**Table 3.** Opioid Prescriptions During the First 12 Weeks After Total Knee Arthroplasty

Variable	MM-R Group, n=70	MM-LB Group, n=70	*P* Value
Initial prescription MME	630 (420-630)	218 (200-225)	<0.0001
Patients with ≥1 refill prescriptions, n (%)	44 (62.9)	37 (52.9)	0.231
Number of refill prescriptions	1 (1-2.5)	2 (1-3)	0.266
Total MME for refill prescriptions	630 (450-1,155)	525 (350-900)	0.443
Total MME for initial and refill prescriptions	803 (630-1,380)	420 (225-810)	<0.0001

Note: Data are presented as median (interquartile range) unless otherwise indicated.

MME, morphine milligram equivalent; MM-LB, multimodal-liposomal bupivacaine; MM-R, multimodal-ropivacaine.

### KOOS and PROMIS-29 Scores

Table 4 shows group, time, and group by time interaction effects for the 12-week postoperative KOOS subscale scores and the PROMIS-29 pain intensity and pain interference scores. Statistically significant group and time effects were observed for all variables. As shown in Table 5, the MM-LB group had statistically significantly better KOOS symptoms, pain, function in activities of daily living, and quality of life scores and PROMIS-29 pain interference scores at 2 weeks (*P*<0.05 for all) compared with the MM-R group. We found no statistically significant between-group differences in any of the outcome measures at the 12-week time point.

**Table 4.** Patient-Reported Outcomes 12 Weeks After Total Knee Arthroplasty

			Fixed Effects		
Outcome	MM-R Group, n=70	MM-LB Group, n=70	Group	Time	Group × Time
KOOS subscale			***P* Value**		
Symptoms	56.5 (2.3)	63.3 (2.1)	0.030	<0.0001	0.184
Pain	53.6 (2.5)	65.3 (2.2)	0.001	<0.0001	0.136
Function in activities of daily living	58.2 (2.6)	66.2 (2.4)	0.025	<0.0001	0.193
Quality of life	34.9 (3.4)	44.3 (2.9)	0.040	<0.0001	0.128
PROMIS-29 score					
Pain intensity	5.2 (0.4)	4.2 (0.3)	0.035	<0.0001	0.601
Pain interference	62.6 (1.2)	57.9 (1.0)	0.005	<0.0001	0.519

Notes: Outcome scores are presented as least squares means (standard error of the mean) adjusted for age and preoperative score and include the 2-week and 12-week data. The actual number of patients who completed outcome surveys at each follow-up visit is reported in Table 5.

KOOS, Knee injury and Osteoarthritis Outcome Score; MM-LB, multimodal-liposomal bupivacaine; MM-R, multimodal-ropivacaine; PROMIS-29, Patient-Reported Outcomes Measurement Information System.

**Table 5.** Patient-Reported Outcomes at 2 and 12 Weeks After Total Knee Arthroplasty by Treatment Group

Outcome	n	MM-R Group	MM-LB Group	*P* Value
KOOS symptoms				
2 weeks	71	50.0 (2.8)	59.2 (3.0)	0.030
12 weeks	80	63.0 (3.3)	67.4 (2.4)	0.301
KOOS pain				
2 weeks	70	44.0 (3.0)	60.2 (3.3)	0.001
12 weeks	78	63.3 (3.6)	70.5 (2.6)	0.119
KOOS function in activities of daily living				
2 weeks	70	48.1 (3.1)	60.0 (3.3)	0.012
12 weeks	77	68.2 (3.7)	72.4 (2.7)	0.366
KOOS quality of life				
2 weeks	64	21.0 (3.8)	34.7 (3.8)	0.014
12 weeks	78	48.8 (4.1)	53.8 (3.1)	0.339
PROMIS-29 pain intensity				
2 weeks	67	6.4 (0.4)	5.2 (0.4)	0.056
12 weeks	81	4.0 (0.5)	3.2 (0.3)	0.201
PROMIS-29 pain interference				
2 weeks	65	66.6 (1.5)	61.0 (1.5)	0.013
12 weeks	77	58.5 (1.8)	54.8 (1.2)	0.101

Notes: Outcome scores are presented as least squares means (standard error of the mean) adjusted for age and preoperative score. The actual number of patients who completed outcome surveys at each follow-up visit is reported in each row as n.

KOOS, Knee injury and Osteoarthritis Outcome Score; MM-LB, multimodal-liposomal bupivacaine; MM-R, multimodal-ropivacaine; PROMIS-29, Patient-Reported Outcomes Measurement Information System.

### Length of Stay

Approximately three-quarters of patients (75.7%, 106/140) were discharged from the hospital on their surgery day, with 19.3% (27/140) spending 1 night in the hospital and 5.0% (7/140) spending ≥2 nights. No statistically significant difference was found in the percentage of patients with a length of stay ≥2 nights by group (*P*=0.602).

### Complications

No medical or surgical complications were reported in the medical records for any of the patients.

## DISCUSSION

In this retrospective cohort study, implementation of a new multimodal pain management protocol resulted in a significant reduction of prescribed opioids without compromising pain and function. Patients who received multimodal analgesia that included liposomal bupivacaine–based regional analgesia reported significantly less pain 2 weeks after surgery while being prescribed significantly less opioids for their initial prescription than patients who received ropivacaine-based regional analgesia. At 12 weeks after TKA, both groups reported a similar level of pain, although the MM-LB group had been prescribed significantly fewer opioids during this period. These findings suggest that the MM-LB group achieved better or equivalent pain control while receiving fewer opioids than the MM-R group at 2 and 12 weeks after TKA. Our findings are consistent with and expand upon a previous retrospective study that reported lower opioid consumption and better pain control during the first 3 days following TKA in patients who received liposomal bupivacaine–based vs ropivacaine-based ACB.^11^

Because all patients in our study received preoperative cryoneurolysis, we cannot separate the effects of the cryoneurolysis intervention from the effects of regional analgesia. However, a large retrospective study that examined opioid prescribing in TKA patients who received preoperative cryoneurolysis^26^ and the present study provide some indirect evidence that both preoperative cryoneurolysis and MM-LB are important components of pain management following TKA. In the retrospective cohort study by Urban et al, patients who received preoperative cryoneurolysis plus MM-R had a lower mean MME for the discharge prescription and a lower cumulative MME at week 6 (660 and 894, respectively) than the control group (1,154 and 1,406, respectively).^26^ In our study, patients who received preoperative cryoneurolysis and MM-LB had a median MME at discharge of 218 and a median cumulative MME at 12 weeks of 420; both values are considerably lower than those reported for the preoperative cryoneurolysis plus MM-R group in the Urban et al study,^26^ suggesting that both preoperative cryoneurolysis and the use of regional liposomal bupivacaine contribute to lower opioid requirements after TKA.

Although randomized clinical trials should be conducted to confirm the results of our study, we believe sufficient scientific evidence and clinical experience support the routine use of both preoperative cryoneurolysis and regional liposomal bupivacaine as part of multimodal pain management for patients undergoing TKA, particularly considering the extremely high burden of opioids, the importance of optimizing patient quality of care and satisfaction, and the cost benefits of achieving faster ambulation and hospital discharge following TKA. Our clinic routinely performs preoperative cryoneurolysis and administers regional liposomal bupivacaine in the context of a multimodal analgesia regimen for patients whose insurance covers these procedures. In a previous study, we demonstrated that the multimodal pain protocol received by the MM-LB group allowed the majority of TKA patients to recover from surgery without the use of any opioids after hospital discharge.^27^ Given that opioid-free TKA is achievable for opioid-naïve patients, we urge the orthopedic community to advocate for more equitable access to opioid-sparing interventions such as preoperative cryoneurolysis, which Medicaid and some commercial payers do not cover.

Several limitations of this study should be noted. First, the study's retrospective nature precludes inferences of causality, and the use of a single site and surgeon limits generalizability. Second, because the study groups were not randomly assigned and treatments were not blinded, results may have been influenced by selection bias, although 70 consecutively treated patients before and after the change in regional analgesia were included. Third, outcomes could possibly be related to some unmeasured differences between groups rather than the change in regional analgesia. Fourth, because the new treatment protocol consisted of 2 simultaneous practice changes (switching from ropivacaine to liposomal bupivacaine for the ACB and adding a liposomal bupivacaine–based iPACK), determining the relative contributions of both components and whether there are additive or synergistic benefits of using liposomal bupivacaine with ACB and iPACK is not possible. Fifth, the standard discharge opioid prescription MME was reduced after the introduction of MM-LB, confounding interpretation of the data to some extent. We note that patients in the MM-LB group could have requested more opioids from the surgeon at any time but were not more likely to request an opioid refill than the MM-R group. This fact, combined with lower pain scores in the MM-LB group at 2 weeks, suggests that pain was well controlled in the MM-LB group despite receiving a lower MME initial prescription. Finally, opioid consumption was based on filled prescriptions and not actual use. A strength of using Louisiana Board of Pharmacy Prescription Monitoring Program data is that the database captures all opioids prescribed by all providers during the study period.

## CONCLUSION

As the number of TKAs performed annually in the United States is expected to increase, developing opioid-sparing and recovery-enhancing strategies is vital. Our study suggests that a multimodal pain regimen that includes preoperative cryoneurolysis and regional analgesia (ACB/iPACK) with liposomal bupivacaine may improve pain during the 2 weeks after surgery and achieve equivalent pain control at 12 weeks post-TKA while reducing opioid requirements compared with traditional regional analgesia (ACB block with ropivacaine). The benefits of these combined interventions should be evaluated in randomized clinical trials and, if confirmed, should be incorporated into clinical practice guidelines.

## Notes

*This article meets the Accreditation Council for Graduate Medical Education and the American Board of Medical Specialties Maintenance of Certification competencies for Patient Care, Medical Knowledge, and Practice-Based Learning and Improvement.*

## References

[1] Lowry V, Ouellet P, Vendittoli PA, Carlesso LC, Wideman TH, Desmeules F. Determinants of pain, disability, health-related quality of life and physical performance in patients with knee osteoarthritis awaiting total joint arthroplasty. Disabil Rehabil. 2018;40(23):2734-2744. doi: 10.1080/09638288.2017.1355412 28728444

[2] Gwam CU, Mohamed NS, Etcheson JI, Changes in total knee arthroplasty utilization since the implementation of ACA: an analysis of patient-hospital demographics, costs, and charges. J Knee Surg. 2020;33(7):636-645. doi: 10.1055/s-0039-1683926 30912105

[3] Sloan M, Premkumar A, Sheth NP. Projected volume of primary total joint arthroplasty in the U.S., 2014 to 2030. J Bone Joint Surg Am. 2018;100(17):1455-1460. doi: 10.2106/JBJS.17.01617 30180053

[4] Wunsch H, Wijeysundera DN, Passarella MA, Neuman MD. Opioids prescribed after low-risk surgical procedures in the United States, 2004-2012. JAMA. 2016;315(15):1654-1657. doi: 10.1001/jama.2016.0130 26978756 PMC4837043

[5] Elmallah RK, Chughtai M, Khlopas A, Pain control in total knee arthroplasty. J Knee Surg. 2018;31(6):504-513. doi: 10.1055/s-0037-1604152 28719941

[6] Bendtsen TF, Moriggl B, Chan V, Børglum J. Basic Topography of the saphenous nerve in the femoral triangle and the adductor canal. Reg Anesth Pain Med. 2015;40(4):391-392. doi: 10.1097/AAP.0000000000000261 26079358

[7] Jæger P, Zaric D, Fomsgaard JS, Adductor canal block versus femoral nerve block for analgesia after total knee arthroplasty: a randomized, double-blind study. Reg Anesth Pain Med. 2013;38(6):526-532. doi: 10.1097/AAP.0000000000000015 24121608

[8] Li D, Yang Z, Xie X, Zhao J, Kang P. Adductor canal block provides better performance after total knee arthroplasty compared with femoral nerve block: a systematic review and meta-analysis. Int Orthop. 2016;40(5):925-933. doi: 10.1007/s00264-015-2998-x 26452678

[9] Chen CM, Yun AG, Fan T. Efficacy of liposomal bupivacaine versus ropivacaine in adductor canal block for total knee arthroplasty. J Knee Surg. 2022;35(1):96-103. doi: 10.1055/s-0040-1713114 32583397

[10] Hungerford M, Neubauer P, Ciotola J, Littleton K, Boner A, Chang L. Liposomal bupivacaine vs ropivacaine for adductor canal blocks in total knee arthroplasty: a prospective randomized trial. J Arthroplasty. 2021;36(12):3915-3921. doi: 10.1016/j.arth.2021.08.017 34556382

[11] Lakra A, Grosso M, Jennings EL, Cooper HJ, Shah RP, Geller JA. Improved pain control with adductor canal block using liposomal bupivacaine after total knee replacement: a retrospective cohort study. Arthroplast Today. 2019;5(3):325-328. doi: 10.1016/j.artd.2019.04.008 31516976 PMC6728435

[12] Schroer WC, Diesfeld PG, LeMarr AR, Morton DJ, Reedy ME. Does extended-release liposomal bupivacaine better control pain than bupivacaine after total knee arthroplasty (TKA)? A prospective, randomized clinical trial. J Arthroplasty. 2015;30(9 Suppl):64-67. doi: 10.1016/j.arth.2015.01.059 26117072

[13] Jain RK, Porat MD, Klingenstein GG, Reid JJ, Post RE, Schoifet SD. The AAHKS Clinical Research Award: liposomal bupivacaine and periarticular injection are not superior to single-shot intra-articular injection for pain control in total knee arthroplasty. J Arthroplasty. 2016;31(9 Suppl):22-25. doi: 10.1016/j.arth.2016.03.036 27113945

[14] Bagsby DT, Ireland PH, Meneghini RM. Liposomal bupivacaine versus traditional periarticular injection for pain control after total knee arthroplasty. J Arthroplasty. 2014;29(8):1687-1690. doi: 10.1016/j.arth.2014.03.034 24793570

[15] Thobhani S, Scalercio L, Elliott CE, Novel regional techniques for total knee arthroplasty promote reduced hospital length of stay: an analysis of 106 patients. Ochsner J. 2017;17(3):233-238. 29026354 PMC5625980

[16] Albrecht E, Wegrzyn J, Dabetic A, El-Boghdadly K. The analgesic efficacy of iPACK after knee surgery: a systematic review and meta-analysis with trial sequential analysis. J Clin Anesth. 2021;72:110305. doi: 10.1016/j.jclinane.2021.110305 33930796

[17] Wang F, Ma W, Huang Z. Analgesia effects of IPACK block added to multimodal analgesia regiments after total knee replacement: a systematic review of the literature and meta-analysis of 5 randomized controlled trials. Medicine (Baltimore). 2021;100(22):e25884. doi: 10.1097/MD.0000000000025884 34087830 PMC8183733

[18] Horner G, Dellon AL. Innervation of the human knee joint and implications for surgery. Clin Orthop Relat Res. 1994;(301):221-226. 8156678

[19] iovera° User Guide. Pacira Pharmaceuticals, Inc. Accessed May 5, 2025. https://www.ioverapro.com/user-guide

[20] Ilfeld BM, Preciado J, Trescot AM. Novel cryoneurolysis device for the treatment of sensory and motor peripheral nerves. Expert Rev Med Devices. 2016;13(8):713-725. doi: 10.1080/17434440.2016.1204229 27333989

[21] Zhou L, Kambin P, Casey KF, Mechanism research of cryoanalgesia. Neurol Res. 1995;17(4):307-311. doi: 10.1080/01616412.1995.11740333 7477749

[22] Trescot AM. Cryoanalgesia in interventional pain management. Pain Physician. 2003;6(3):345-360. 16880882

[23] Roos EM, Lohmander LS. The Knee injury and Osteoarthritis Outcome Score (KOOS): from joint injury to osteoarthritis. Health Qual Life Outcomes. 2003;1:64. doi: 10.1186/1477-7525-1-64 14613558 PMC280702

[24] Hays RD, Spritzer KL, Schalet BD, Cella D. PROMIS®-29 v2.0 profile physical and mental health summary scores. Qual Life Res. 2018;27(7):1885-1891. doi: 10.1007/s11136-018-1842-3 29569016 PMC5999556

[25] Prescrption Monitoring Program (PMP) Information. Louisiana Board of Pharmacy. Accessed May 16, 2025. pharmacy.la.gov/page/prescription-monitoring-program-pmp-information

[26] Urban JA, Dolesh K, Martin E. A multimodal pain management protocol including preoperative cryoneurolysis for total knee arthroplasty to reduce pain, opioid consumption, and length of stay. Arthroplast Today. 2021;10:87-92. doi: 10.1016/j.artd.2021.06.008 34286056 PMC8280475

[27] Bronstone AB, Leonardi C, Brown J, Crabb R, Dasa V. Multimodal opioid-sparing analgesia for total knee arthroplasty: results from a retrospective case series of 40 patients. JOEI. 2022;3(1). doi: 10.60118/001c.33296

